# The Essentials of Vitamin and Mineral Supplementation After Total Gastrectomy

**DOI:** 10.1007/s12029-025-01240-w

**Published:** 2025-06-06

**Authors:** Rachael C. Lopez, Amber F. Gallanis, Jeremy L. Davis

**Affiliations:** 1 Clinical Center Nutrition Department, National Institutes of Health, Bldg 10, RM 9 C442, 10 Center Dr, MSC 1078, Bethesda, MD 20892-1078 USA; 2 Surgical Oncology Program, National Cancer Institute, National Institutes of Health, Bethesda, MD USA; 3 Division of Surgical Oncology, Department of Surgery, University of Maryland School of Medicine, Baltimore, MD 21201 USA

**Keywords:** Total gastrectomy, Micronutrient supplementation, Postgastrectomy syndrome

Total gastrectomy (TG), whether performed to treat or prevent gastric cancer, imparts numerous long-term sequelae, notably, life-altering nutrition-related complications, including micronutrient deficiencies [1, 2]. Current NCCN guidelines recommend micronutrient supplementation after gastrectomy; however, specific information on vitamin dosing and formulation is notably absent and providers are instead directed to follow local practice guidelines [3]. As a result, postgastrectomy nutrition guidelines vary significantly across institutions, with many providers recommending a chewable multivitamin, such as the Flintstones™ vitamin, which lacks the nutritional supplementation required for individuals post-TG. A general lack of standardization highlights the need for evidence-based recommendations to prevent micronutrient deficiencies, and their consequences, in individuals who undergo TG for prevention or treatment of gastric cancer. Micronutrient deficiencies impart significant physical and mental health consequences such as muscle weakness, cognitive changes, impaired immune function, severe fatigue, and osteoporosis (Table 1). Therefore, consequences of inadequate supplementation can negatively impact patients’ productivity, financial stability, and quality of life [1].

**Table 1:** Consequences of micronutrient deficiencies after bariatric surgery

Micronutrient	Consequences	Prevalence
Vitamin B12	Megaloblastic anemia, peripheral neuropathy, slowed mentation, dementia	Up to 20% after RYGB
Copper	Fatigue, weakness, anemia, osteoporosis, nerve damage, muscle weakness	Up to 20% after RYGB
Zinc	Hair loss, taste changes/loss of taste, diarrhea, infertility, impaired immune function, impaired healing	Up to 40% after RYGB
Vitamin B1 (Thiamin)	Weight loss, anorexia, confusion, short-term memory loss, muscle weakness, congestive heart failure, peripheral neuropathy, Wernicke’s encephalopathy, confusion, coma, death	Up to 49% after bariatric surgery
Iron	Severe fatigue, weakness, lightheadedness, pallor, brittle nails, shortness of breath, feeling cold, reduced academic or work performance/productivity, decreased physical capacity, anemia	Up to 55% after RYGB
Folate	Megaloblastic anemia, weakness, fatigue, difficulty concentrating, oral ulcers, intestinal symptoms	Up to 65% after bariatric surgery
Vitamin A	Night blindness, xerophthalmia, fatigue	Up to 70% after bariatric surgery
Calcium and Vitamin D	Rickets, osteopenia, osteoporosis, fracture, tooth decay, weak nails, muscle spasms	Up to 100% after bariatric surgery

*RYGB*, Roux-en-Y gastric bypass

Although developed for individuals undergoing bariatric surgery, the American Society of Metabolic and Bariatric Surgery (ASMBS) guidelines after gastric bypass with Roux-en-Y reconstruction (RYGB) are applicable to patients who undergo TG [4]. This is because of the similarly restrictive and malabsorptive nature of both operations [4]. Both RYGB and TG reduce absorption of micronutrients due to (1) decreased or lack of stomach acid, which decreases absorption of calcium and iron, (2) decreased or lack of intrinsic factor, which decreases absorption of vitamin B12, (3) bypass of the duodenum and proximal jejunum, which decreases absorption of iron, calcium, thiamine, among other micronutrients, and (4) delayed mixing of food with bile and pancreatic juices, causing decreased absorption of fat-soluble vitamins [5]. Lifelong nutritional supplementation with a specialty post-bariatric formulation should be applied to both individuals who elect for bariatric surgery and TG [6].

TG reduces nutrient absorption via similar mechanisms as RYGB; however, the risk of micronutrient deficiencies is higher after TG due to the complete absence of both stomach acid and intrinsic factor, rapid transit of food due to loss of a gastric reservoir and decreased oral tolerance due to postgastrectomy symptoms. The long-term, deleterious impact of postgastrectomy symptoms on patients’ nutrition status cannot be ignored [1]. Previous research has shown that 2 years after TG, 94% of patients experienced at least one chronic sequelae such as dumping syndrome, bile reflux, and/or micronutrient deficiency [1]. Due to the complete loss of stomach function after TG, patients frequently report absence of hunger cues, extreme early satiety, often feeling full after just a few bites of food, nausea, early dumping syndrome, late dumping syndrome, and bile reflux [7]. Additionally, the authors’ clinical experience is that oral micronutrient supplements can worsen symptoms such as nausea, constipation, and bile reflux. Prescribing multiple, individual supplements to meet nutritional needs after TG results in a heavy pill burden, which may contribute to poor adherence to supplementation guidelines and subsequently increases the risk of deficiency [8]. Therefore, the optimal micronutrient regimen should focus on adequate supplementation, while limiting pill burden.

Following total gastrectomy, it is the authors’ practice to prescribe a specialized, iron-containing multivitamin that meets ASMBS guidelines after RYGB, with an additional 1200–1500 mg (mg) of calcium citrate daily. Postgastrectomy patients are advised to take calcium in divided doses up to 500 mg each, separately from iron to promote absorption. These specialized multivitamins, commonly referred to as bariatric multivitamins, are commercially available over the counter and provide vitamin B12, vitamin D, zinc, thiamine, folate, iron, among other micronutrients, at doses developed to support adequate absorption in individuals with altered gastrointestinal anatomy after TG and bariatric surgery. In accordance with current practice guidelines, we recommend daily oral supplementation to meet nutritional requirements, with intravenous or intramuscular administration only when oral supplementation is inadequate or not tolerated by the patient [9].

Patient referral to a registered dietitian experienced with postgastrectomy nutritional requirements for guidance before and after TG is strongly recommended [2, 7, 10]. Dietitians and healthcare providers alike must emphasize the need for lifelong multivitamin supplementation in individuals who undergo TG and review appropriate supplementation with each patient. As most dietary supplements are available over the counter and not typically covered by health insurance in the USA, it is imperative that health care providers assess the specific supplements being used. We recommend dietitians verify that supplements meet ASMBS guidelines and have been third-party tested for safety and accuracy, given that multivitamins and nutrient supplements are not regulated by the Food and Drug Administration (FDA). Despite appropriate counseling and vitamin supplementation, individuals without a stomach may still experience micronutrient deficiencies. Therefore, we advocate for lifelong monitoring of micronutrient levels to facilitate early identification and correction of deficiencies if they do occur [1]. Detailed discussion of our institutions’ nutrition education program after TG, including suggested blood tests for micronutrient surveillance and rates of micronutrient deficiencies have been previously described [1, 2, 10].

Standard oral multivitamins, including prenatal vitamins, which are formulated to meet the Recommended Daily Allowance for healthy individuals with an intact gastrointestinal tract, are *ineffective* at preventing or treating deficiencies long-term after RYGB or TG (Table 2) [11, 12]. Unsurprisingly, a standard pediatric chewable multivitamin, such as Flintstones™ vitamin, formulated to meet the micronutrient needs of children with intact gastrointestinal tracts, does not prevent or treat deficiencies after total gastrectomy. A simple comparison of the micronutrient content in a standard Flintstones™ brand pediatric multivitamin compared to the ASMBS guidelines following RYGB, emphasizes the discrepancy in appropriate nutrient supplementation (Table 2). For example, the pediatric multivitamin provides 0.6 mg of thiamine compared to the ASMBS guidelines recommended dose of ≥ 12 mg. Thiamine deficiency can be life-threatening, resulting in congestive heart failure and/or Wernicke’s encephalopathy, making it essential to follow established ASMBS micronutrient guidelines. One example of a specific bariatric multivitamin that meets ASMBS guidelines and has been third-party tested provides 20 mg of thiamine (Table 2); other products that fulfill these criteria may also be considered appropriate. For patients to thrive after TG, the onus is on the provider to equip them with this crucial knowledge about their micronutrient needs. Therefore, it is equally essential for health care providers caring for postgastrectomy patients to provide thorough, evidence-based instruction on the appropriate supplementation and need for strict adherence to lifelong vitamin and mineral supplementation [13].

**Table 2:** Comparison of ASMBS Guidelines for micronutrient supplementation after RYGB with a bariatric formulated multivitamin and with Flintstones™ brand pediatric multivitamin

Micronutrient	ASMBS guidelines for daily supplementation after RYGB to prevent deficiency [9]	ProCare Health™ bariatric multivitamin with iron^a^	Flintstones™ with iron chewable multivitamin
Vitamin A	5000–10,000 international units	10,000 international units (75% as beta carotene)	400 mcg = ~ 1870 IU (10% as beta-carotene)
Vitamin D	3000 international units or enough to maintain 25 (OH) D level > 30 ng/mL	3000 international units	600 international units
Vitamin E	15 mg	60 international units (40 mg)	7 mg
Vitamin K	90–120 mcg	120 mcg	0 mcg
Thiamin	> / = 12 mg daily	20 mg	0.6 mg
Folate	400–800 ug oral daily from multivitamin	800 mcg dietary folate equivalents	200 mcg dietary folate equivalents (120 mcg folic acid)
Vitamin B12	350–1000 mcg daily or 1000 mcg intramuscular monthly	1000 mcg	1.2 mcg
Iron	> / = 45–60 mg elemental iron daily, taken separately from calcium supplements	45 mg	18 mg
Zinc	8–22 mg Supplementation should contain a ratio of 8–15 mg supplemental zinc per 1 mg of copper to minimize risk of copper deficiency	16 mg	5 mg
Selenium	Unknown but likely higher than 100 mcg/day	100 mcg	0
Copper	2 mg	2 mg	0
Calcium	1200–1500 mg, given in divided doses	n/a	n/a

^a^This is provided as one example of an acceptable product and does not imply endorsement. Other brands of bariatric multivitamins that meet ASMBS guidelines and that are third party tested for accuracy may also be acceptable

*ASMBS*, American Society for Metabolic and Bariatric Surgery; *RYGB*, Roux-en-Y gastric bypass

Current management guidelines recommend total gastrectomy for the treatment of certain proximal gastric cancers and prophylactic total gastrectomy at age 20 to 30 years for individuals at risk of hereditary diffuse gastric cancer with germline *CDH1* pathogenic or likely pathogenic (P/LP) variants [14]. While the goal of therapeutic and prophylactic TG is to prolong life, lifelong micronutrient supplementation with the appropriate, bariatric formulated vitamins is essential to prevent complications from deficiencies. TG is an uncommon operation and not without many risks and long-term sequelae, many of which can severely affect quality of life and result in debilitating complications. We advocate for the application of evidence-based guidelines on micronutrient supplementation after RYGB to TG to prevent the adverse consequences of micronutrient deficiencies postgastrectomy. Simply put, *Yabba Dabba Don’t* prescribe Flintstones™ vitamins after total gastrectomy.

## Data Availability

No datasets were generated or analysed during the current study.
